# School nutrition laws in the US: do they influence obesity among youth in a racially/ethnically diverse state?

**DOI:** 10.1038/s41366-021-00900-8

**Published:** 2021-07-20

**Authors:** Emma V. Sanchez-Vaznaugh, Mika Matsuzaki, Paula Braveman, Maria Elena Acosta, Kelsey Alexovitz, James F. Sallis, Karen E. Peterson, Brisa N. Sánchez

**Affiliations:** 1 Department of Public Health, San Francisco State University, San Francisco, CA USA; 2 Center for Health Equity, University of California, San Francisco, CA USA; 3 Department of International Health, Johns Hopkins Bloomberg School of Public Health, Baltimore, MD USA; 4 Department of Epidemiology and Biostatistics, Dornsife School of Public Health, Drexel University, Philadelphia, PA USA; 5 Division of Behavioral Medicine, Department of Family and Medicine and Public Health, University of California, San Diego, CA USA; 6 Department of Nutritional Sciences, School of Public Health, University of Michigan, Ann Arbor, MI USA

**Keywords:** Obesity, Risk factors

## Abstract

**Background/objectives:**

Little is known about the separate or combined effects of state and national nutrition policies regulating food and beverages in schools on child overweight/obesity (OV/OB) and related racial/ethnic disparities. We investigated the influence of school nutrition policies enacted in California, independently and in combination with the United States’ national policy “Healthy Hunger Free Kids Act” (HHFKA) on childhood OV/OB and racial/ethnic disparities.

**Subjects/methods:**

An interrupted time series design was used with data from 12,363,089 child-level records on 5th- and 7th-graders in California public schools to estimate sex- and racial/ethnic-specific time trends in OV/OB prevalence during three periods: before the California nutrition policies (2002–2004); when only California policies were in effect (2005–2012); and when they were in effect simultaneously with HHFKA (2013–2016).

**Results:**

Before the state’s policies, OV/OB prevalence increased annually among children in most subgroups. Improvements in OV/OB trends were observed for almost all groups after the California policies were in effect, with further improvements after the addition of HFFKA. The total change in annual log-odds of OV/OB, comparing the periods with both state and federal policies versus no policies, ranged from −0.08 to −0.01 and varied by grade, sex, and race/ethnicity. Within each sex and grade, the greatest changes were among African-American (−0.08 to −0.02, all *p* < 0.05) followed by Latino children (−0.06 to −0.01, all *p* < 0.05). Although disparities narrowed among these groups versus White children after the dual policy period, disparities remained large.

**Conclusions:**

State and national nutrition policies for schools may have contributed to containing the upward trend in childhood OV/OB and racial/ethnic OV/OB disparities within California. However, sizable OV/OB prevalence and disparities persist. To end the epidemic, promote healthy weight and increase health equity, future efforts should strengthen state and national policies to improve food quality in schools, particularly those serving populations with the highest OV/OB prevalence.

Childhood obesity has increased dramatically around the world, prompting concerns and calls for interventions to curb the epidemic, given the negative health consequences associated with obesity[1, 2]. Over the past two decades, policies to improve diet and prevent childhood overweight/obesity (OV/OB) have received considerable attention in many countries around the world [3–6]. In the United States (U.S.), thirty percent of children are living with either overweight or obesity. African-American and Latino children are more likely to live with OV/OB than their White peers [7, 8], underscoring the urgent need to identify effective interventions to reduce and/or eliminate racial/ethnic health disparities-one of the U.S. national public health priorities. In response to the U.S. child obesity epidemic, local, state and federal policies have sought to improve the nutritional content of foods and beverages in schools. Starting in the 2004–2005 school year, California, the most populous state in the U.S., was one of the first to implement policies for “competitive” foods and beverages (CF&B), so called because they are sold to students in schools, and therefore compete with federally supported school meal programs. CF&B policies were nationally adopted a decade later through the US Department of Agriculture’s (USDA) “Smart Snacks in School” rule. To address concerns over nutritional quality and potential link to obesity [9], the national Healthy Hunger Free Kids Act (HFFKA) policy updated the nutrition standards for school meals starting in the 2012–2013 school year.

Previous research examined the influence on obesity of either state and/or local CF&B policies or policies for school meals, primarily in the United States [10–12] but also in other countries such as Canada and China [13, 14]. While research has associated state and local CF&B policies with reduced overweight and/or obesity among students, other studies [10–12], many with weaker designs have not observed these associations [15, 16] or reported mixed evidence [17]. Considering federal initiatives, quasi-experimental studies have observed lower increases in BMIz scores among school meal participants than non-participants [18] and yearly declines in obesity among children in poverty post-HHKFA relative to obesity increases prior to improvements in school meal standards [19]. Studies examining nutrition standards similar to HHFKA [20] found USDA-school meal standard components were associated with reduced OV/OB [21], especially among participants of the school meal program [20].

No study has investigated the combined population-level influences of state policies for competitive food and beverages and national policies for school meals overall or on racial/ethnic disparities. The present study examined the influence on childhood OV/OB overall and OV/OB disparities of (a) the California school nutrition policies alone, and (b) in combination with the federal policy for school meals.

## Methods

### State and federal school nutrition policies

Effective in July 2004, the state of California school nutrition policy SB677, restricted the sale of beverages in elementary and middle schools to: water, milk, 100% juice, juice drinks with at least 50% juice (no added sweeteners) and sport drinks. Sugary beverages not listed in the law were banned from sale during the school day. Effective January 2006, California SB965 updated beverage standards for elementary and middle schools to include: drinking water with no added sweeteners, vegetable-based drinks that are composed of no less than 50% vegetable juice and have no added sweeteners, limited the fat content of milk to ≤2%, and allowed non-dairy milks. Effective July 1, 2004, California SB677 set nutrition standards for snacks in elementary schools only, however in July 2007, SB12 set improved nutrition and portion size standards for competitive foods in grades K-12 statewide, limiting the percent of total calories from fat to no more than 35% by weight, from saturated fats to ≤10%, and sugar content in snacks to ≤35% by weight. SB 490 banned the use of *trans*-fats in competitive foods for grades K-12 effective in 2009, and SB80 banned *trans*-fats and fried foods in school meals. The federal “Smart Snacks” policy, effective 2014–2015, set standards for competitive foods and beverages that are generally similar to California’s policies.

Effective in 2012-2013, the national HHFKA policy (enacted in 2010) mandated new nutrition standards for meals offered as part of the school meals program: (a) requiring daily availability of and variety of fruits, vegetables over the school week (Dark green, Red/Orange, Legumes) and whole grain-rich foods; (b) limiting milk to fat free or low fat; (c) reducing sodium levels, saturated and trans-fats; and (d) ensuring age-appropriate portion sizes. The state’s CF&B and the HHFKA policy are complementary in that the former (except for SB80) targeted items sold to students outside the school meals, whereas the latter targeted improvement in the nutrition standards for school meals. CF&B policy details are in Supplemental Table A.

### Sources of data and study variables

Child-level anthropometry and physical fitness are collected in schools each year from February to March via the Fitnessgram battery for California public school students in 5th-, 7th- and 9th-grades, including height, weight, physical fitness measures, age, sex, race/ethnicity and grade. Students in the respective grades are required to take as much of the test as possible. To account for differences in school- and district-level characteristics, the 2002-2016 Fitnessgram data were merged with school-level information from public databases, and using school geocodes, merged with census information for the 2000 and 2010 Censuses, and the 2015 American Community Survey.

#### Student-level variables

Child-level OV/OB status was obtained by: calculating body mass index (BMI) as weight in kilograms divided by height in meters squared, classifying students as OV/OB if their age- and sex-specific BMI was ≥ the 85th percentile of the CDC’s 2000 reference distribution. Other student-level variables included sex, age (in years), race/ethnicity (classified as African-American, Asian, Latino and White), and fitness level (classified as meeting fitness standards vs not, based on the Cooper Institute’s guidelines for the time to run 1 mile) or the PACER when time to run 1 mile was unavailable.

#### School-level variables

School characteristics might influence the policies’ implementation and BMI. Thus, school size (enrollment), and racial/ethnic composition were considered. Schools were classified as “majority” for one of the aforementioned four major racial/ethnic groups if ≥50% of the students enrolled were in that group; otherwise, the school was classified as “other or no majority.” School’s (continuous) and school districts’ (in quartiles) annual proportion of students eligible for free or reduced price meals were used, as socioeconomic factors are associated with overweight/obesity and student-level socioeconomic information is unavailable in Fitnessgram.

#### School neighborhood socioeconomic variables

Annual median household income of the residents within the school’s census tracts and the proportion of residents aged ≥25 who completed ≥16 years of education were used as socio-economic indicators. Linear interpolation between the values of these characteristics in 2000, 2010, and 2015 were used to obtain time-varying socioeconomic characteristics for each school and each year.

### Study population

This study was restricted to African-American, Asian, Latino, and White 5th- and 7th-graders who attended California public schools during 2002–2016; the study focuses on these students as the timing and content of nutrition policies differed for high schools. Of the 12,863,306 child-level records from these groups available in the Fitnessgram database during 2002–2016, 12,384,247 (96%) were from schools required to comply with the state policies, thus eligible for inclusion in the analysis. Of those, 12,363,089 (99.8%) students in 7879 schools had complete data on age and sex, thus, a fixed a priori sample size for this study. Among children with complete demographics, 10% had missing data on one or more variables of interest. Hence, five imputed datasets were obtained using multivariate imputation by chained equations using the R mice package, so that analysis included all children eligible for the study. The data for this study were provided by the CDE. Therefore, the study was reviewed by and received approval from the Committee for the Protection of Human Subjects of the California’s Office of Statewide Health Planning and Development. The author’s institutions exempted the study from review given its use of secondary data.

### Statistical analyses

Following descriptive statistics, an interrupted time series design [22] and multilevel logistic regression with OV/OB as the dichotomous outcome estimated and compared the slope of the trend lines fitted to the yearly log-odds of OV/OB before and after the policies. These estimated slopes (hereafter “trends”) describe whether the likelihood of OV/OB increased, decreased, or stayed unchanged from one year to the next within a given period. Changes in trends (e.g., from increasing to decreasing trend) capture the gradual accrual of the policies’ influence from one period to another. Trends were estimated using three terms: time in years since 2002 captured the slope prior to the California policies, a linear spline term with a knot placed at 2005 estimated changes in slopes following the California policies, and a spline term with a knot at 2013 estimated further/additive changes in slopes associated with HHFKA. Interactions between these terms and race/ethnicity examined the policies’ influence on disparities. A single model coefficient (*t* test) or combinations of model coefficients (chi-squared) were used to evaluate statistical significance of the results. Testing combinations of coefficients is necessary given the ITS design (e.g., slope of trends after the policy is the sum of the pre-policy slope plus the change in the slope). The tests of coefficients rely on the assumption of normality of the sampling distribution for the coefficients, which is satisfied given the study’s large sample size. Multivariate normal random intercepts and slopes (for year since 2002 and spline terms), with unstructured covariance matrices, at the level of schools and school-districts were used to account for clustering among students and for the possibility of heterogeneity [23] of trends at these levels. The models were adjusted for student-, school-, and district-level covariates and were grade-and-sex stratified given documented age and sex adiposity differences. The same models were fitted to all five imputed datasets, and the model results were combined using standard formulae to combine results from multiple imputation analysis. Model results were used to estimate and plot annual prevalence. These prevalence estimates were compared to counterfactual prevalence: estimates for 2005–2016 obtained using linear projection of the pre-policy (2002–2004) trends, which is supported by linear increases observed for this age group in national data [7]. Code to generate model results can be obtained from the corresponding author by email request. Results are plotted with confidence intervals for each group included; in these plots, differences in the precision of confidence intervals across groups is driven by sample size differences across groups. A set of results are provided for descriptive purposes, thus the plots exclude confidence intervals to aid readability.

## Results

**Fig. 1:**
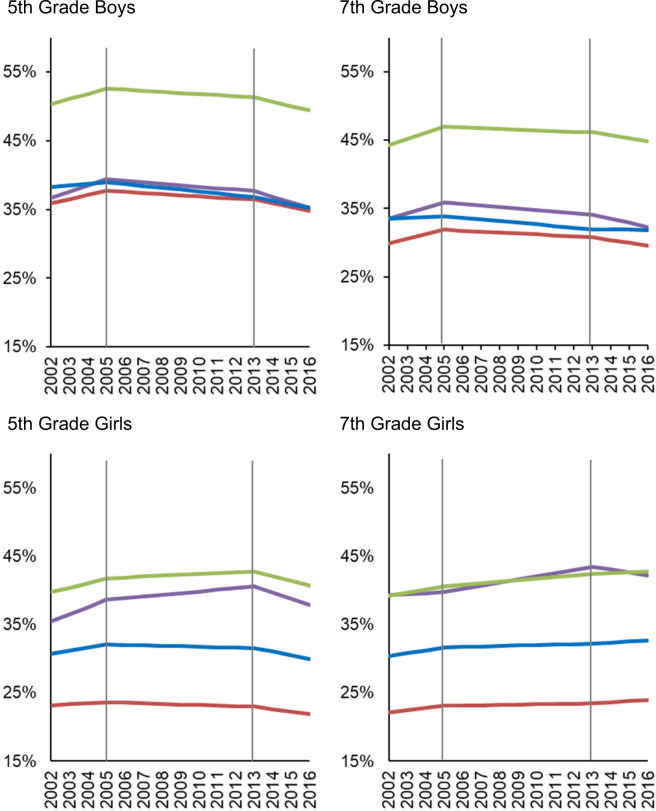
Yearly prevalence of overweight/obesity during the study period. For each grade and sex, the panels show yearly estimates of overweight/obesity prevalence for each racial/ethnic group before (2002–2004) and after the California Nutrition Policies (2005–2012), and after the Federal Policy for School Meals (HHFKA) Took Effect (2013–2016). Estimates are based on models using annual California student-level Fitnessgram data from 2002 to 2016, adjusted for student, school, and school-neighborhood covariates. Vertical lines depict inflection points when policies are assumed to accrue effects. The color lines represent each of the racial/ethnic groups: Purple = African American; Red = Asian; Green = Latino; Blue = White.

Of the 12,363,089 student records, 7.6% were African-American, 9.3% Asian, 53.4% Latino and 29.7% White. The characteristics of children are in Supplementary Table B. The prevalence of OV/OB was lowest among Asians, and highest among Latino and African-American students overall and show variation over the study period (Fig. 1).

### Changes in overweight/obesity prevalence trends by grade and racial/ethnic group

**Fig. 2:**
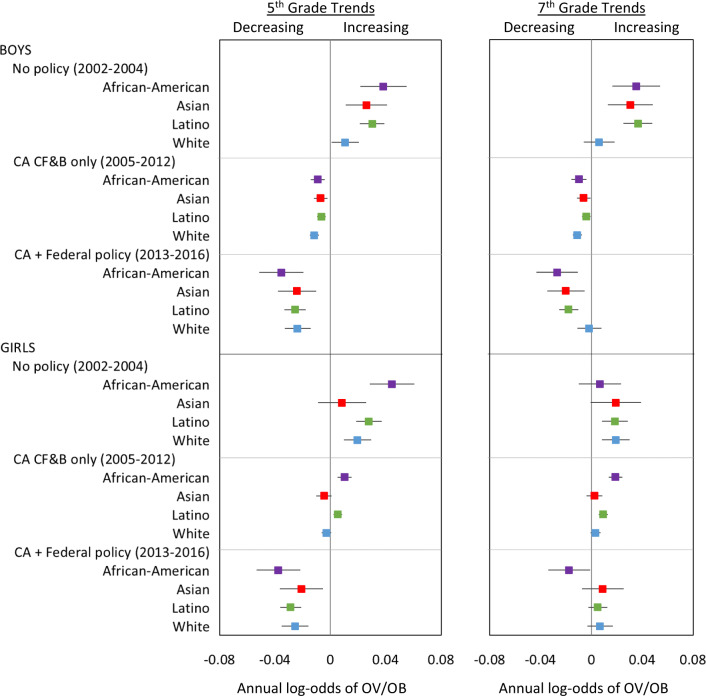
Trends in overweight/obesity within each policy period. For each grade-, sex, and racial/ethnic group, the panels show annual change in log-odds of overweight/obesity along with 95% confidence intervals, before (2002–2004) and after the California Nutrition Policies (2005–2012), and after the Federal Policy for School Meals (HHFKA) took effect (2013–2016). Estimates are based on models using annual California student-level Fitnessgram data from 2002 to 2016, adjusted for student, school, and school-neighborhood covariates. The point estimates and confidence intervals show the annual log-odds of OV/OB within each time period, representing the extent to which the OV/OB prevalence was increasing (positive slope), decreasing (negative slope), or plateaued (slope not significantly different from zero).

Prior to any policy (2002–2004), OV/OB increased significantly each year among all subgroups except for four groups: 5th-grade Asian girls, 7th-grade Asian, African American girls and White boys, for whom overweight/obesity increased though not-significantly (Fig. 2). When only the CA nutrition policies were in effect (2005–2012), OV/OB trends changed significantly in a favorable direction for nearly all groups. In 2005–2012, OV/OB decreased annually among 5th and 7th grade boys in all racial/ethnic groups as well as 5th grade Asian and White girls (though declines were not significant in the latter two groups). Overweight/obesity increased slightly though non-significantly among 7th grade Asian and White girls, and increased significantly, but at a slower pace among African American and Latina girls in both grades.

After the HHFKA went into effect (2013–2016), further favorable changes in OV/OB trends were observed, with significant annual declines among all 5th-graders across racial/ethnic and sex groups and among 7th-grade African-American children (both sexes), Asian, and Latino boys but not significantly in 7th grade White boys. OV/OB trends plateaued among 7th-grade Asian, Latina and White girls (Fig. 2).

**Fig. 3:**
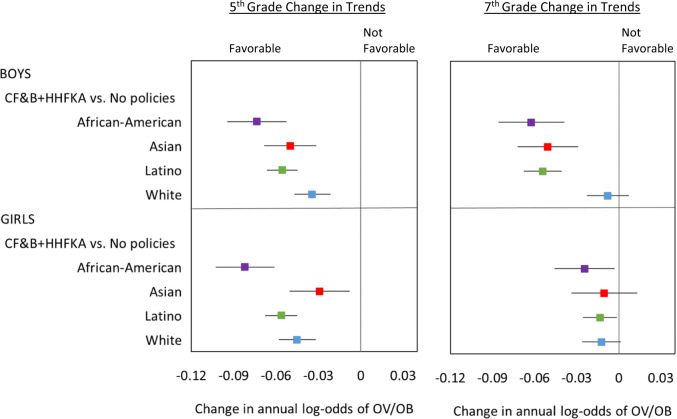
Cumulative change in overweight/obesity trends. For each grade, sex and racial/ethnic group, the figure shows changes in OV/OB trends, comparing the period when both state and federal policies were simultaneously in place (2013–2016) to the period when no policies were in place (2002–2004). Estimates are based on models using California student‐level Fitnessgram data 2002‐2016, adjusted for student, school, and school‐neighborhood covariates. The point estimates and confidence intervals show the change in the annual the log‐odds of OV/OB comparing periods as indicated, representing the extent to which the prevalence trends changed in a favorable direction (negative change in slope), or not favorable direction (positive change in slope), or remained unchanged (difference in slope not significantly different from zero).

In sum, when both the state and federal nutrition policies overlapped (2013–2016), OV/OB trends changed significantly for all but three subgroups—from prior increases to declines or plateaus—compared to the period without policies (2002–2004). The magnitude of the total change in annual log-odds of OV/OB ranged from −0.03 to −0.08 among 5th-graders, and −0.01 to −0.06 among 7th-graders and varied by race/ethnicity (Fig. 3). The greatest changes were among 5th-grade African-American and Latino children, 7th-grade African-American and Latino boys, and Asian boys in both grades.

#### Racial/ethnic disparities in overweight/obesity

**Fig. 4:**
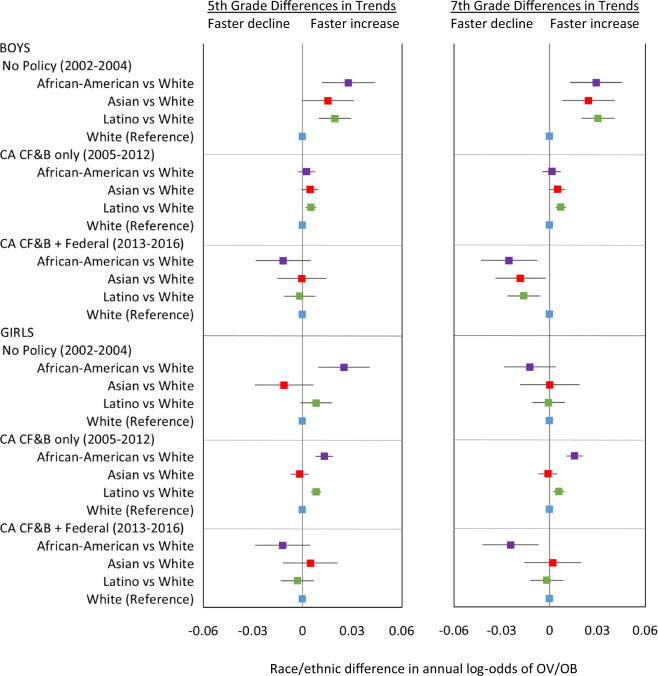
Disparities in OV/OB trends within each policy period. For each grade and sex group, figure shows racial/ethnic overweight/obesity disparities in the trends before (2002–2004) and after the California Nutrition Policies (2005–2012), and after the Federal Policy for School Meals (HHFKA) took effect (2013–2016). Based on models using annual California student-level Fitnessgram data from 2002 to 2016, adjusted for student, school and school-neighborhood covariates.

**Fig. 5:**
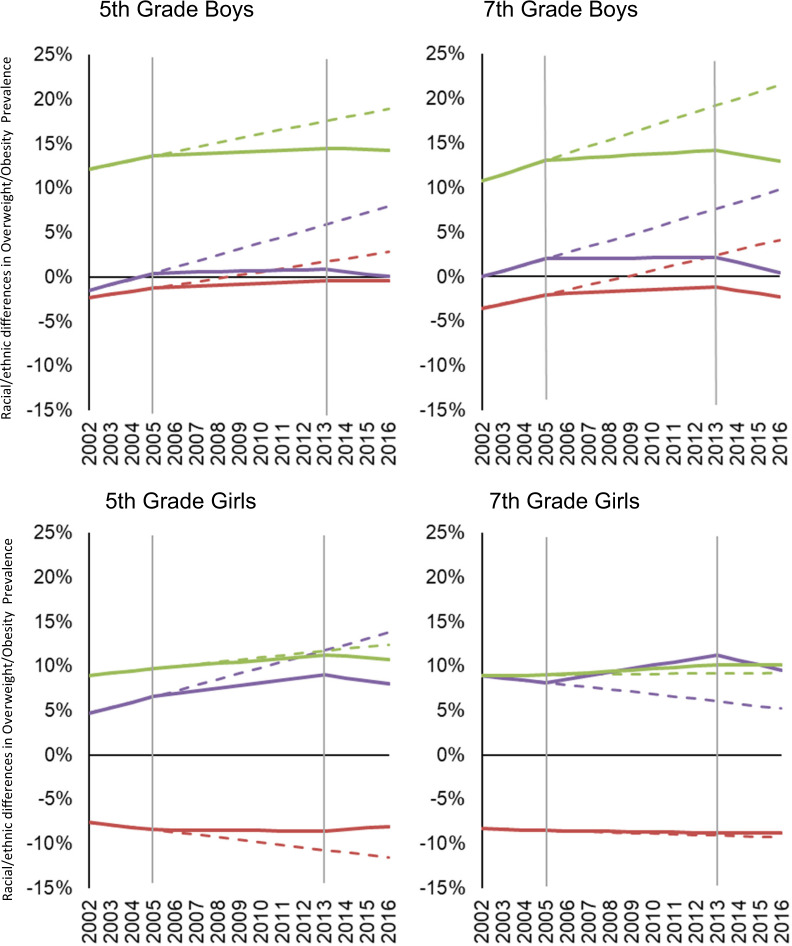
Disparities in OV/OB prevalence during the study period. For each grade and sex group, panels show racial/ethnic disparities in overweight/obesity prevalence. The dashed lines assume pre-policy trends remain unabated; solid lines show disparities estimates before (2002–2005) and after the California Nutrition Policies (2005–2012), and after the Federal Policy for School Meals (HHFKA) took effect (2013–2016). Based on models using annual California student-level Fitnessgram data from 2002 to 2016, adjusted for student, school and school- neighborhood covariates. Horizontal Line at 0% indicates white peers are the reference. Vertical lines depict inflection points when policies are assumed to accrue effects. Negative percents mean the Asian students had lower OV/OB prevalence compared with their White peers in respective grade. The color lines represent each of the racial/ethnic groups: Purple = African American; Red = Asian; Green = Latino.

**Fig. 6:**
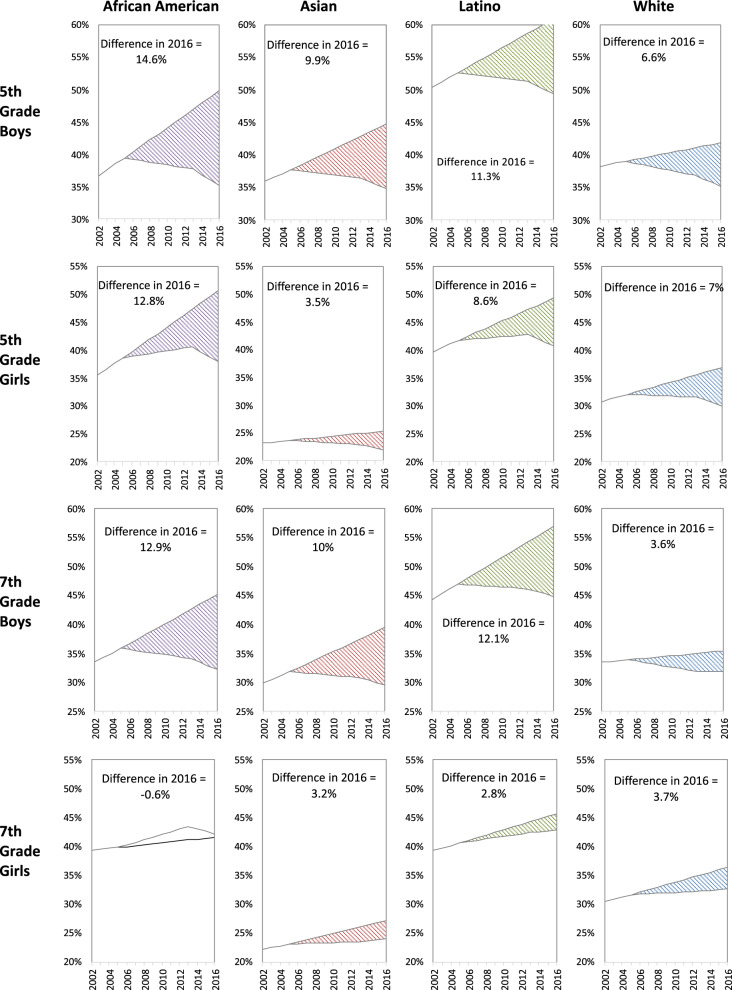
Comparison of estimated and counterfactual overweight/obesity prevalence with and without the policies. For each sex-, grade-, and racial/ethnic-group, the panels show overweight/obesity prevalence estimated using models fitted with the policies in place (solid line) and a counterfactual prevalence assuming trends in the pre-policy period (2002–2004) continue (dashed lines). Shaded area represents the difference in 2016 prevalence without versus with the policies in place.

Prior to the California policies (2002–2004), OV/OB disparities widened among 5th-grade African-Americans (boys and girls) and Latino boys, and 7th-grade African-American and Latino boys versus their White counterparts within the sex-grade combinations, given that OV/OB prevalence increased significantly faster among these groups versus their White counterparts (Fig. 4 and Fig. 5). Although Asian boys in both grades experienced lower OV/OB prevalence, the rate of OV/OB increased was faster than their White peers. Disparities were neither increasing nor decreasing among 5th-grade Asian and 7th-grade African-American, Asian and Latina versus White girls within the respective grades. After the California CF&B policies went into effect (2005–2012), OV/OB disparities ceased to increase among 5th and 7th-grade African-American boys, disparities increased though at a slower pace among 5th-grade African-American girls and Latino boys in both grades and increased among 7th grade African American girls versus White counterparts within the respective sex-grade combinations. Latina vs White girls disparities increased in both grades though the magnitude was small (Figs. 4 and 5). When state and federal policies were simultaneously in effect (2013–2016), OV/OB declined faster (non-significantly) among 5th-grade African-Americans and Latinos (both sexes) and Asian boys, and decreased significantly faster among 7th-grade African-Americans (both sexes), Latino and Asian boys, compared to their White peers in respective grade-sex combinations (Fig. 4). During this dual policy period (2013–2016), OV/OB disparities slightly narrowed among 5th- and 7th-grade African-Americans in both sexes and 7th-grade Latino boys. The OV/OB African-American/White gap closed among 7th-grade boys, though that disparity was small. Asian children had lower or similar OV/OB prevalence than their White peers. Overall, the potential influence of the policies in preventing overweight/obesity is critically important: assuming OV/OB trends continued without the policies, the 2016 OV/OB prevalence could have been 4–15% higher among boys or 1–13% higher among girls, depending on race/ethnicity (Fig. 6).

## Discussion

The California school nutrition policies and the federal policy governing nutrition standards for school meals were associated with favorable changes in OV/OB—either a downward trend or a leveling off of prior increases—among all groups studied. The combined policies also were associated with halting the widening of racial/ethnic OV/OB disparities we observed in the pre-policy period and/or slightly narrowing those during the dual policy period among 5th-grade African-Americans in both sexes, 7th-grade African-American and Latino boys compared with their White peers within respective grades.

This is the first study to investigate the combined influences of state and national school nutrition policies on population-level trends in OV/OB and to examine additive effects of these policies on four major racial/ethnic groups, including changes in racial/ethnic disparities within a diverse state in the U.S. The novel findings were generated using an interrupted time series design [22]—one of the strongest to evaluate non-randomized policy interventions [24]. The changes in population-level OV/OB trends and disparities associated with these policies are of high population-level and public health significance [25]. The downward shifts in OV/OB by the end of the study period suggest the policies could have been responsible for reductions of >10% OV/OB prevalence in several subgroups. Small population-level shifts in OV/OB associated with school policies have the potential to reduce cardiometabolic disease related deaths later in life [26].

The policy effects on childhood OV/OB are plausible through dietary pathways, which the policies intended to modify. State and/or school-district level policies regulating CF&B similar to those in the state of California have been associated with reduced in-school availability of CF&B, and reduced student consumption of sugary beverages [13, 16], lower caloric intake overall, and calories from solid fats and added sugars [13, 27, 28] and unhealthy snacks [27, 29] in schools [10, 12]. Additionally, a study found California students reported lower intake of fat, sugar and calories after implementation of CF&B policies than students in states without such laws [27]. A CF&B district-level policy similar to the state’s policy was associated with reduced student consumption of soda and fried foods among students [30] in California. Evaluations of the U.S. HHFKA policy in nationally representative data have also found significant increases in the nutritional quality of school meals two years post-implementation [31–33]. Studies in various states, including California found no changes or declines in student fruit consumption, increased consumption of the entrée meal, and vegetables [34, 35], lower energy [34] and caloric intake[34], and increased nutrient quality of meals students selected [36] from pre-to post HHFKA implementation. This evidence, combined with the complementary roles of the state and federal policies in regulating different types of foods/beverages, suggests that California children, particularly those who started school after the state’s policies, were exposed to increasingly better school food environments.

The containment of the upward trend in racial/ethnic disparities during the period with no policies, and/or the reduced OV/OB gaps we observed after the combined policies took effect, particularly HHFKA are small although encouraging. These favorable findings are consistent with our a priori hypothesis and the fact that HHFKA targets socially disadvantaged children. Previous studies observed smaller obesity disparities between school lunch participants and non-participants in U.S. states with standards similar to HHKFA [20], a diminished rising trend in overweight/obesity among school lunch participants [37], and a declining obesity trend among children in poverty post-HHFKA [19]. Although these studies did not directly examine policy effects on racial/ethnic OV/OB disparities, together, results from these and the present study provide evidence to support the goals of the federal school meals policy in addressing diet quality disparities [11], and overall child health, including the prevention of OV/OB and the reduction of related disparities among low income, Latino and African-American vs White children.

In the context of the present study, it is plausible that the combined state and federal school nutrition policies curtailed previously widening racial/ethnic OV/OB disparities via dietary pathways as well. A national study observed state-level CF&B policies were associated with fewer servings of soda among African-American versus White students [16]. The absence of sugary beverages in middle schools was associated with lower OV/OB prevalence among Latino but not other students [21]. The higher propensity of African-Americans to purchase or consume sodas sold in school venues [38] may have been impacted by the CF&B policies in the state of California. Moreover, greater obesity declines were observed among Latinos and African-Americans than among White students [39, 40], following nutrition policy interventions in two school districts. An alternative explanation for the observed reductions in disparities is that families of African-American and Latino students included here may have been exposed to nutrition interventions in other environments during the study period. This is unlikely to fully explain the findings, as children spend an average of 7 h in school on a daily basis and consume up to fifty percent of their daily calories at these settings [41, 42]. Additionally, we are not aware of statewide family/home nutrition interventions specific to non-white children that coincided with the timing of the state and federal school nutrition policies.

The likelihood that the combination of policies had a favorable impact on OV/OB is supported by: the strength of the study design; documented dietary pathways, the coincident changes in OV/OB trends with the timing of the policies; and the consistency of the trend changes among all groups after both policies were in place versus when no policies were in effect. Nevertheless, isolating policy effects is always challenging because policies are seldom implemented as randomized experiments. We considered alternative explanations to our study findings; namely, the potential roles of: secular trends in OV/OB; physical activity; non-school dietary quality; and other interventions such as the Robert Wood Johnson Foundation’s (RWJF) $500 million childhood obesity initiative. Secular trends are an unlikely alternative explanation. National data from 1999–2016 showed a linear increase in OV/OB among adolescents [7], yet our study shows evidence of favorable changes in OV/OB trends in California. Changes in physical activity could have played a role in the changes in OV/OB trends we observed; for example, due to targeted initiatives [43], such as the national Let’s Move campaign or the RWJF’s initiative. This is unlikely to fully explain our study results because we adjusted for students’ fitness status, an objective indicator related to both overall physical activity [44], and to quantity and quality of physical education [45]. Improvements in food environments outside of schools may have driven the observed changes in OV/OB trends, but it is doubtful that this contributed significantly to the findings, for example, among African-American and Latino children, given detrimental changes in the fast food environment near-schools attended by these student populations [46].

The study limitations include our inability to unequivocally conclude the results are solely attributable to the policies as exposure to them was non-randomized, though we used the interrupted time series design—which is appropriate when there is no clear control group—and adjusted for time-varying covariates. While the federal policy appeared to have additive effects, these findings could be due to a persistent or lagged effect of the California CF&B policies, yet we could not estimate the federal policy’s effect absent the California policies. Data on policy implementation were unavailable, precluding dose-response assessments. Previous research [47] involving 19 California schools found adherence to the state’s CF&B standards improved between 2005 and 2008. By 2008, 61% and 100% of elementary schools adhered to the food and beverage standards respectively; 63% and 81% of middle schools adhered to the standards. Similarly, a high proportion of California schools (90%) were certified in 2014 as meeting the school meal standards [48]. We were unable to verify the quality of the outcome data, though a Texas study noted high validity and reliability of BMI based on teacher collected height and weight measures compared with those gathered by trained specialists [49]. The investigators did not have data from prior to 2002, thus the pre-CF&B policy trend based on four data points may increase uncertainty in the estimates of trend/changes. Nevertheless, the sizable nature of the data for this study enabled a retrospective evaluation of the effects of both policies combined on measured overweight/obesity status and on racial/ethnic overweight/obesity disparities.

This study provides new evidence to support calls to improve, not weaken, school nutrition policies [50, 51] and environments more broadly [3], as well as to focus on populations with highest OV/OB prevalence [52]. It is unsurprising that improvements in school food environment was insufficient to reverse the obesity epidemic or substantially reduce racial/ethnic disparities in the California context. The study’s results and the continuing high prevalence of childhood OV/OB underscore the need to strengthen the quality of food and beverages in schools [50, 53, 54], and to reduce barriers to policy implementation, including lack of equipment, staff training, and food costs [42, 48]. Existing or new policies could address these barriers by allocating additional state and/or federal resources, collecting evaluation data and monitoring policy compliance [55]. The present study findings can help inform childhood obesity prevention policies in countries that have implemented school nutrition policies such as in Europe, Norway, Switzerland [5] and Mexico, as well as countries where childhood obesity has not yet reached epidemic proportions.

Children are exposed to numerous and complex food environments [56]. Strategic interventions could consider food systems and related economic incentives [57], regulate unhealthy food advertising to children [58] to enhance all food environments [3] including the provision and promotion of healthy alternatives. Widespread racial/ethnic and socioeconomic disparities in the built environment, require explicit attention in efforts to reduce/eliminate persistent OV/OB disparities [52], including increasing the quantity, quality and availability of healthy foods, snacks and beverages, reducing unhealthy foods in racial/ethnic and low income communities [52, 59] and improving social and economic resources [52].

State and federal school nutrition policies may have contributed to curbing the pre-policy upward trend in childhood OV/OB and the widening racial/ethnic disparities in the state of California, the most populous and one of the most racially/ethnically diverse in the United States. However, the prevalence of OV/OB remains high, and racial/ethnic OV/OB disparities persist. To reverse the childhood obesity epidemic and eliminate related disparities, future efforts should strengthen state and federal school nutrition policies, and target child populations with the highest prevalence.

## Supplementary information

Supplemental Material
