# Correction to: School nutrition laws in the US: do they influence obesity among youth in a racially/ethnically diverse state?

**DOI:** 10.1038/s41366-021-00932-0

**Published:** 2021-08-16

**Authors:** Emma V. Sanchez-Vaznaugh, Mika Matsuzaki, Paula Braveman, Maria Elena Acosta, Kelsey Alexovitz, James F. Sallis, Karen E. Peterson, Brisa N. Sánchez

**Affiliations:** 1 Department of Public Health, San Francisco State University, San Francisco, CA USA; 2 Center for Health Equity, University of California, San Francisco, CA USA; 3 Department of International Health, Johns Hopkins Bloomberg School of Public Health, Baltimore, MD USA; 4 Department of Epidemiology and Biostatistics, Dornsife School of Public Health, Drexel University, Philadelphia, PA USA; 5 Division of Behavioral Medicine, Department of Family and Medicine and Public Health, University of California, San Diego, CA USA; 6 Department of Nutritional Sciences, School of Public Health, University of Michigan, Ann Arbor, MI USA

**Keywords:** Obesity, Risk factors

Correction to: *International Journal of Obesity* 10.1038/s41366-021-00900-8, published online 20 July 2021

The original version of this article unfortunately contained a mistake in the legends of Figs. 1 and 5. The additional sentence to fig. legend 1 is: The color lines represent each of the racial/ethnic groups: Purple = African American; Red = Asian; Green = Latino; Blue = White. The additional sentence to fig. legend 5 is: The color lines represent each of the racial/ethnic groups: Purple = African American; Red = Asian; Green = Latino. The journal apologizes for the omission of the legends. The original article has been corrected per author’s request.

