# Supplementary material for: School nutrition laws in the US: do they influence obesity among youth in a racially/ethnically diverse state?
Source: Int J Obes (Lond). 2021 Jul 20;45(11):2358–68. doi: 10.1038/s41366-021-00900-8 (PMC8528713; doi:10.1038/s41366-021-00900-8)
Supplement: Supplementary file 1 — Supplemental Material [file 41366_2021_900_MOESM1_ESM.docx]

**Supplemental Material**

School Nutrition Laws in the US: Do they Influence Obesity Among Youth in a Racially/Ethnically Diverse State?

[Table A. Comparisons of key standards for “Competitive” foods and beverages (CF&B): California* vs. Federal policy (the latter known as Smart Snacks in Schools). 2](#_Toc70691514)

[Table B. Characteristics of California Fifth and Seventh-grade public school students and the schools they attended, (2002-2016), by Race or Ethnicity, and Overall 3](#_Toc70691515)

[References for Table A 4](#_Toc70691516)

Table A. Comparisons of key standards for “Competitive” foods and beverages (CF&B): California* vs. Federal policy (the latter known as Smart Snacks in Schools).

| **STANDARDS** | **California CF&B**  **Policies^1-4^** | | | | | **Federal Policy**  **for Smart Snacks^5^** | | | | | |
| --- | --- | --- | --- | --- | --- | --- | --- | --- | --- | --- | --- |
|  | **ES**** | **MS** | | **HS** | | **ES** | | **MS** | | **HS** | |
| **FOODS^** (✓=standard in place; UR=unregulated) | | | | | | | | | | | |
| ***SNACKS*** |  | | | | | | | | | | |
| Total Calories | <175 | | <250 | | <250 | | <200 | | <200 | | <200 |
| <35% calories from fat | ✓ | | ✓ | | ✓ | | ✓ | | ✓ | | ✓ |
| <10% calories from saturated fat | ✓ | | ✓ | | ✓ | | ✓ | | ✓ | | ✓ |
| <35% sugar by weight | ✓ | | ✓ | | ✓ | | ✓ | | ✓ | | ✓ |
| Zero trans fats (<0.5 grams) | ✓ | | ✓ | | ✓ | | ✓ | | ✓ | | ✓ |
| <200 mg sodium | UR | | UR | | UR | | ✓ | | ✓ | | ✓ |
| ***FULL MEALS OR ENTREES*** |  | | | | | | | | | | |
| All of the above (except calories) | UR | | ✓ | | ✓ | | ✓ | | ✓ | | ✓ |
| Calories | UR | | <400 | | <400 | | <350 | | <350 | | <350 |
| <480 mg sodium | UR | | UR | | UR | | ✓ | | ✓ | | ✓ |
| <4 grams of fat per 100 calories | UR | | ✓ | | ✓ | | UR | | UR | | UR |
| Effective start date | 2004 | | 2007 | | 2007 | | 2014/15 | | 2014/15 | | 2014/15 |
| **BEVERAGES^^** (symbols as above, and in addition: X = item banned) | | | | | | | | | | | |
| ***ALLOWED/BANNED THROUGHOUT SCHOOL DAY***^a^ |  | | | | | | | | | | |
| Water (plain or carbonated) | Allowed | | Allowed | | Allowed | | Allowed | | Allowed | | Allowed |
| Milk and milk alternatives | Allowed | | Allowed | | Allowed | | Allowed | | Allowed | | Allowed |
| Flavored milk (i.e., chocolate milk) | UR | | UR | | UR | | Allowed | | Allowed | | Allowed |
| 100% Fruit or vegetable juice | Allowed | | Allowed | | Allowed | | Allowed | | Allowed | | Allowed |
| Fruit or vegetable-based drinks, no added sweeteners^b^ | Allowed | | Allowed | | Allowed | | Allowed | | Allowed | | Allowed |
| Electrolyte replacement beverages (ERB) | X | | Allowed | | Allowed | | X | | X | | Allowed |
| Calorie-free flavored water (plain or carbonated) | X | | X | | X | | X | | X | | Allowed |
| Other beverages with <40 calories/8 oz. | X | | X | | X | | X | | X | | Allowed |
| All other beverages not listed | X | | X | | X | | X | | X | | X |
| ***NUTRITION STANDARDS FOR BEVERAGES*** |  | | | | | | | | | | |
| Water: no added sweeteners | ✓ | | ✓ | | ✓ | | ✓ | | ✓ | | ✓ |
| Milk^c^: fat content | <2% | | <2% | | <2% | | <1% | | <1% | | <1% |
| Flavored milk: fat content | UR | | UR | | UR | | non-fat | | non-fat | | non-fat |
| Fruit or vegetable juice: % of fruit/vegetable allowed | 100% | | 100% | | 100% | | 100% | | 100% | | 100% |
| Fruit/vegetable-based drinks, no added sweeteners:  max volume of 100% juice^d^ | >50% | | >50% | | >50% | | UR | | UR | | UR |
| ERB: <42 grams added sweetener/20oz. | X | | ✓ | | ✓ | | X | | X | | X |
| Calorie-free flavored water only: <5 calories/8 oz. | X | | X | | X | | X | | X | | ✓ |
| Other beverages: <40 calories/8 oz. | X | | X | | X | | X | | X | | ✓ |
| All beverages: caffeine free | UR | | UR | | UR | | ✓ | | ✓ | | UR |
| ***SIZE LIMITS (IN FLUID OUNCES)*** |  | | | | | | | | | | |
| Water (plain or carbonated) | UR | | UR | | UR | | UR | | UR | | UR |
| Milk and milk alternatives | UR | | UR | | UR | | <8 oz | | <12 oz | | <12 oz |
| Flavored milk (i.e., chocolate milk) | UR | | UR | | UR | | <8 oz | | <12 oz | | <12 oz |
| Fruit or vegetable juice | UR | | UR | | UR | | <8 oz | | <12 oz | | <12 oz |
| Fruit/vegetable-based drinks, no added sweeteners | UR | | UR | | UR | | <8 oz | | <12 oz | | <12 oz |
| Electrolyte replacement beverages (ERB) | X | | UR | | UR | | X | | X | | <12oz^e^ |
| Calorie-free flavored water (plain or carbonated) | X | | X | | X | | X | | X | | <20 oz^e^ |
| Other beverages with <40 calories/8 oz. | X | | X | | X | | X | | X | | <12 oz |
| Effective start date | 2004 | | 2004 | | 2007 | | 2014/15 | | 2014/15 | | 2014/15 |
| ^*^ The scope and effect dates of the California beverage and food policies varied for elementary, middle, and high schools. See footnotes below.  ^**^ SB 12 established grade levels as follows: (a) **“Elementary school**” means a public school that maintains any grade from kindergarten to grade 6, inclusive, but no grade higher than grade 6. (b) “**Middle school**” means any public school that maintains grade 7 or 8, 7 to 9, inclusive, or 7 to 10, inclusive. (c) “High school” means any public school maintaining any of grades 10 to 12, inclusive.  ^^^ **Food policies**: In California SB 677, effective July 1, 2004, set nutrition standards for snacks in elementary schools only.^1^ Effective in 2007, SB 12 set improved nutrition standards in schools K-12.^3^ Effective in 2009, SB 490 added standards that applied to trans fats for K-12.^~~4~~^ The federal policy for Smart Snacks took effect in the academic year 2014-15.  ^^^^ **Beverage policies:** In California SB 677 went into effect on July 1, 2004, setting beverage standards for elementary, middle and junior high schools.^1^ SB 965 updated those standards for elementary and middle schools to include: drinking water with no added sweetener, vegetable-based drinks that are composed of no less than 50 percent vegetable juice and have no added sweetener and limited milk to fat content and milk types: two percent-fat, one percent-fat, nonfat, as well as soy milk, rice milk, and other similar nondairy milk, standards that went into effect on January 1, 2006. Additionally, starting in 2007, SB 965 extended beverage standards into high schools, where 50% of beverages had to meet standards, and by July 2009 100% of beverage had to adhere to specifiedstandards.  ^a^ Beverages that do not comply are allowed to be sold for school fundraisers and events, such as dances; these vary by school level.  ^b^ Fruit or vegetable-based drinks diluted with water (incl. carbonated), no added sweeteners  ^c^ “Milk” means cow's or goat's milk that: (1) Contains Vitamin A, Vitamin D, and at least 25 percent of the FDA-established Daily Value (DV) for calcium per 8 ounces. (2) Contains no added sweeteners exceeding 28 grams of total sugars per 8 ounces. (3) Is two-percent fat milk, one-percent fat milk, or nonfat milk. “Non-dairy milk” alternative (e.g., rice milk, soy milk) means a beverage that: (1) Contains Vitamin A, Vitamin D and at least 25 percent of the DV for calcium per 8 ounces, (2) Contains no added sweeteners exceeding 28 grams of total sugars per 8 ounces, and(3) Contains no more than 5 grams of fat per 8 ounces.  ^d^ SB 677 and SB 965 aimed to increase juice quality even when diluted with water, by requiring that at least half of the fluid ounces provided in the beverage container must contain 100 percent juice. Per Food and Drug Administration rules, the percent juice must be included on the label.  ^e^ Per federal law, other beverages, including electrolyte replacement beverages, must < 5 calories per 8 fluid oz in maximum serving size of 20 fluid oz or ≤ 40 calories per 8 fluid oz in maximum serving size of 12 fluid oz. | | | | | | | | | | | |

| Table B. Characteristics of California Fifth and Seventh-grade public school students and the schools they attended, (2002-2016), by Race or Ethnicity, and Overall.^a,b,c^ |
| --- |

| **N** | **White 3,677,485** | **Latino 6,607,765** | **Asian 1,143,646** | **African-**  **American 934,193** | **Overall 12,363,089** |
| --- | --- | --- | --- | --- | --- |
| **Sex ( % Female)** | 48.5 | 49.1 | 48.6 | 49.4 | 48.9 |
| **Grade, %** |  |  |  |  |  |
| 5th | 50.2 | 51.8 | 50.5 | 51.2 | 51.2 |
| 7th | 49.8 | 48.2 | 49.5 | 48.8 | 48.8 |
| **Age (Years), %** |  |  |  |  |  |
| 9 | 0 | 0 | 0.1 | 0.1 | 0 |
| 10 | 25.8 | 28.4 | 30.3 | 27.9 | 27.8 |
| 11 | 23.5 | 21.8 | 19.7 | 21.6 | 22.1 |
| 12 | 26.6 | 27.9 | 30 | 28.1 | 27.7 |
| 13 and older | 24.1 | 21.9 | 19.9 | 22.3 | 22.4 |
| **Physical Fitness^d^, %** |  |  |  |  |  |
| Meets Standard | 46.6 | 45.3 | 52 | 42.2 | 46.1 |
| Exceed Standard | 26.6 | 16.6 | 22.5 | 16.8 | 20.1 |
| Needs Improvement | 26.9 | 38.2 | 25.5 | 41 | 33.8 |
| **Overweight/Obese Classification^e^, %** | | | | | |
| 2002 | 30.3 | 47.1 | 27.7 | 38.9 | 38.8 |
| 2003 | 30.6 | 48.1 | 28.6 | 39.7 | 39.7 |
| 2004 | 30.5 | 48.3 | 28.2 | 40.2 | 40 |
| 2005 | 31 | 49.6 | 28.3 | 41.3 | 41 |
| 2006 | 30.1 | 48.7 | 28.3 | 41 | 40.5 |
| 2007 | 29.7 | 48.5 | 27 | 41.5 | 40.3 |
| 2008 | 29.6 | 48.5 | 27 | 41.3 | 40.3 |
| 2009 | 29.4 | 48.2 | 26.6 | 40.9 | 40.1 |
| 2010 | 29.7 | 48.3 | 27.2 | 41.4 | 40.3 |
| 2011 | 28.3 | 48.2 | 25.5 | 41.3 | 40.3 |
| 2012 | 28.3 | 47.7 | 25.7 | 40.9 | 39.9 |
| 2013 | 27.4 | 47.1 | 24.7 | 39.7 | 39.1 |
| 2014 | 27.2 | 47.1 | 24.5 | 39.7 | 39.2 |
| 2015 | 27.2 | 47.1 | 24.1 | 39.7 | 39.1 |
| 2016 | 27.5 | 46.7 | 24.2 | 39.3 | 39 |
| **Percent of Enrolled Children who Receive Free or Reduced-Price meals** | | | | | |
| 2002 | 32.9 | 66.9 | 39.1 | 59.6 | 51.8 |
| 2016 | 39.9 | 72.9 | 40.2 | 68.1 | 60.8 |
| **Percent of School Neighborhood^f^ Residents with 16+ Years of Education** | | | | | |
| 2002 | 30.2 | 16.3 | 33.9 | 22.4 | 23.3 |
| 2016 | 38.5 | 21.2 | 42.8 | 26.7 | 28.2 |
| **Median Household Income of Residents in the School Neighborhood^f^ (in USD$1,000)** | | | | | |
| 2002 | 63.1 | 46.0 | 65.7 | 49.5 | 54.0 |
| 2016 | 84.0 | 58.9 | 90.4 | 63.9 | 68.9 |
| ^a^ Children excluded were those: 1) Student whose age is outside of expected age range for a given grade; 2) Race/ethnicity other than White, African American, Asian or Latino.  ^b^ Children can contribute data in more than one grade, therefore the total number per race/ethnicity is the total number of records.  ^c^ Data from the California Fitnessgram, available through the California Department of Education.  ^d^ Physical Fitness is defined as scoring within the Cooper Institute's Fitnessgram healthy fitness zone according to performance on the 1-mile run or walk; when children had missing data on 1-mile run, their 1 mile run time was imputed based on their performance on the PACER test, using an existing equivalency chart between 1-mile run time and PACER.  ^e^ Overweight/Obese is defined as age- and sex-specific body mass index (BMI) calculated as weight in kg divided by height in meters squared at or above the 85th percentile of the reference distribution.  ^f^ School neighborhood is defined as the school’s census tract. | | | | | |

Table A References

1. California Senate Bill No. 677. The California Childhood Obesity Prevention Act. 2003-2004. An act to amend Section 49431 of, and to add Section 49431.5 to, the Education Code, relating to schools. Available at <https://leginfo.legislature.ca.gov/faces/billNavClient.xhtml?bill_id=200320040SB677>
2. California Senate Bill No. 965. Pupil nutrition: beverages. 2005-2006. An act to amend Section 49431.5 of the Education Code, relating to pupils. Available at <https://leginfo.legislature.ca.gov/faces/billNavClient.xhtml?bill_id=200520060SB965>
3. California Senate Bill No. 12. School food nutrition. 2005-2006. An act to amend Sections 49430, 49431, 49433.9, and 49434 of, and to add Section 49431.2 to, the Education Code, relating to pupils. Available at <https://leginfo.legislature.ca.gov/faces/billNavClient.xhtml?bill_id=200520060SB12>
4. California Senate Bill No. 490. Pupil nutrition: trans fats. 2007-2008. An act to add Section 49431.7 to the Education Code, relating to pupil nutrition. Available at <https://leginfo.legislature.ca.gov/faces/billNavClient.xhtml?bill_id=200720080SB490>
5. Food and Nutrition Service, US Department of Agriculture. National School Lunch Program and School Breakfast Program: Nutrition Standards for All Foods Sold in School as Required by the Healthy, Hunger-Free Kids Act of 2010; Interim Final Rule, 7 CFR §210 and 220 (2013).
